# A review of selected Arboviruses during pregnancy

**DOI:** 10.1186/s40748-017-0054-0

**Published:** 2017-10-03

**Authors:** Penélope Saldanha Marinho, Antonio José Cunha, Joffre Amim Junior, Arnaldo Prata-Barbosa

**Affiliations:** 1 Maternidade Escola da Universidade Federal do Rio de Janeiro, Rio de Janeiro, RJ Brazil; 2 Faculdade de Medicina da Universidade Federal do Rio de Janeiro, Rio de Janeiro, RJ Brazil; 3 Instituto D’Or de Pesquisa e Ensino – IDOR, Rio de Janeiro, RJ Brazil; 4 Rua das Laranjeiras, 180 – Laranjeiras, Rio de Janeiro, CEP: 22240-003 RJ Brazil

**Keywords:** Arboviruses, Pregnancy, Dengue, Zika, Chikungunya, Yellow fever, Review

## Abstract

Arboviruses are emerging infectious diseases with the ability to expand geographically and rapidly affect large populations.

The recent epidemic caused by the Zika virus in the Americas and congenital Zika syndrome associated with maternal infection has called out attention to the importance of studying arboviruses during pregnancy.

This is a review on selected arboviruses infections during gestation, including Zika, Chikungunya, Dengue and Yellow Fever viruses. Issues such as historical overview, pathogenesis, transmission, clinical conditions, diagnosis, treatment and prevention are addressed.

## Background

Arboviruses are emerging infectious diseases which have gained importance in the last 50 years due to their ability to expand geographically and rapidly affect large populations. The spread of these diseases in the human population occurs through the introduction of the pathogen into the environment, associated with other factors such as ecological changes and human behavior. As the mosquito vectors breed in standing water, events like heavy rain with flooding and construction of dams are associated with the emergence of infections by arboviruses. In addition, urbanization, migration and international travel are also contributing factors to the transmission of these disease [1, 2].

Recently, the epidemic caused by the Zika virus in the Americas has led the World Health Organization to declare a state of international emergency, providing global visibility to the issue, which has become a public health priority.

The present study consists in a review on arboviruses during gestation, addressing infections caused by the Zika, Chikungunya, Dengue and Yellow Fever viruses.

## History of arboviruses

Arboviruses are thus designated since they are viruses essentially transmitted by arthropods, predominantly mosquitoes. At least 135 types of arboviruses are capable of causing human infection, with the vast majority being RNA viruses [3].

### Zika

The Zika virus was initially isolated in 1947 from Rhesus monkey blood during epidemiological studies on Yellow Fever in the Zika Forest, in Uganda [4]. This virus was first isolated in humans in 1954, in Nigeria [5] and, in 1969, it was identified outside the African continent, in Malaysia, in the *Aedes aegypti* mosquito [6]. Throughout the following years, until 2007, the Zika virus was known to causes sporadic infections in both Africa and Asia.

The first recognized major outbreak occurred on Yap Island, Micronesia, in 2007 [7], followed by a major outbreak from October 2013 to March 2014 in French Polynesia [8, 9].

In 2015, the Zika virus reached the Americas, and was initially identified in Brazil, where a phylogenetic analysis indicated it was the Asian strain of the virus [10]. According to the Pan American Health Organization report of July 2017, cases of autochthonous transmission have been reported at least in 50 other countries or territories of the American continent, totaling more than 200,000 confirmed cases, with approximately 3300 cases of congenital syndrome associated with the Zika infection [11].

### Chikungunya

The Chikungunya virus was reported for the first time in Tanzania in 1952. After the first epidemic, the virus became endemic in Africa. It reached Asia in 1958 and was responsible for several outbreaks in subsequent years [12].

The virus remained restricted to the African and Asian continents until 2013, when it was introduced in the Latin Caribbean and established the first human cycle in the Americas [13]. Subsequently, cases of autochthonous transmission were reported in South and Central America and in Florida, United States of America [14].

In September 2014, the first autochthonous case was confirmed in Brazil, in the northern region. After 7 days, new cases were identified in the northeastern region and, in just 1 month, the Ministry of Health of Brazil had already confirmed 682 cases of this virus infection [15].

### Dengue

Although numerous epidemics with Dengue-like characteristics have been described for centuries, the Dengue virus was first isolated in 1943 in Japan (DENV1) and in 1945 in Hawaii (DENV2) [16].

The spread of its vector, the *Aedes aegypti* mosquito, created an environment conducive to the virus transmission, which reached a pandemic status after World War II [17].

In the Americas, Dengue was controlled through a campaign conducted by the Pan American Health Organization in 1947 to eliminate mosquitoes. However, in the 1970s, the region was reinfested and, since then, the incidence of the disease has increased in the Americas, as well as in other regions [18].

### Yellow fever

Yellow Fever originated in Africa and was brought to the Americas in the seventeenth century as a consequence of the slave trade, and was responsible for the deaths of thousands of people in the 18th and 19th centuries. In 1900, transmission was confirmed through the *Aedes aegypti* mosquito and, in the 1930s, two live attenuated virus vaccines were developed. The combination of extensive vaccination campaigns and vector control significantly reduced the number of cases of this disease [19].

Over the years, outbreaks in Africa and South and Central America continued to occur [20]. In December 2015, an urban Yellow Fever epidemic occurred in Angola and expanded to other locations, and in 2017, an outbreak of the disease affected the Brazilian states of Minas Gerais, Espírito Santo, São Paulo and Rio de Janeiro [21].

## Pathogenesis and transmission

### Mechanical transmission

The Zika, Dengue and Yellow Fever viruses belong to the Flaviviridae family and Flavivirus genus, while the Chikungunya virus belongs to the Togaviridae family and Alphavirus genus. All are transmitted in the urban cycle, mainly by mosquito species *A. aegypti* and *A. alpobictus*.

Arboviruses display a transmission cycle between the mosquito and the human host, with periods of both extrinsic and intrinsic incubation. Extrinsic incubation refers to the period that elapses between the vector infection until the virus spreads to the mosquito’s saliva, which then infects the new host. The intrinsic incubation period refers to the time interval between inoculation in the human host until the virus reaches the bloodstream.

The pathogenesis of arboviruses still remains unknown in several respects, but it is recognized that, upon primary inoculation, viral particles infect target cells such as fibroblasts, monocyte, dendritic cells and host endothelial cells. The viruses then replicate, reaching the regional lymph nodes, and, subsequently, the bloodstream [22].

### Sexual transmission and blood transfusion

Sexual transmission of the Dengue, Chikungunya or Yellow Fever viruses has not been described. However, transmission of the Zika virus through infected semen has already been well established [23]. In addition, regarding the identification of virus RNA in semen, which may persist for more than 60 days after the onset of the symptoms [24], some studies have confirmed the disease in people who have not been in endemic areas but who have had sexual contact with infected persons.

A review of a study of sexually active women of Rio de Janeiro highlights the contribution of sexual transmission of the Zika virus. Women between the ages of 15 and 65 presented 90% more cases recorded in 100,000 women than men of the same age group. This was not repeated in groups <15 years and > 65 years [25].

Arboviruses can also be transmitted by blood transfusion or transplantation of organs or tissues from infected and asymptomatic donors at the time of donation. In the case of Yellow Fever, transmission is also possible during the viremia period after donor vaccination [26–30].

### Maternal-fetal transmission

The occurrence of arboviruses during pregnancy is of additional concern, due to the possibility of vertical transmission and fetal involvement. In 2015, a significant increase in the number of newborns diagnosed with microcephaly in several regions of Brazil presenting a high incidence of Zika virus was observed. Maternal-fetal transmission of the virus was verified during all gestation stages [31–34].

Torres et al. found a vertical transmission rate for the Chikungunya virus ranging from 27.7 to 48.29%, occurring more frequently when maternal viremia coincided with the time of birth. However, the birth route showed no relation to the incidence of congenital disease [35].

Infection of pregnant women with the Dengue virus is associated with the risk of miscarriage, fetal death, prematurity and vertical transmission. Although gestation is considered a risk factor for the clinical course of the disease, no association was found between the severity of the maternal infection and neonatal disease [36, 37].

Few studies on yellow fever are available, but perinatal transmission of the disease has been described [38].

## Clinical conditions

The clinical presentation of these arboviruses during pregnancy is supposed to be similar to the general population. However, there is a lack of studies specifically addressing pregnant women.

### Zika

The incubation period of this disease is of 4 to 5 days. The most frequently reported symptoms are a rash, fever, arthritis or arthralgia, and conjunctivitis. Other symptoms include myalgia, headache, retro-orbital pain, edema and vomiting. It is estimated that only 20% of the people infected with the Zika virus manifest symptoms [39].

In a cohort of pregnant women in Rio de Janeiro, where exanthema was used as the inclusion criterion, in addition to macular or maculopapular exanthema in 100% of the cases, the following symptoms were also observed: pruritus (90%), arthralgia (62%), conjunctivitis (58%), fatigue and malaise (52%), myalgia (41%) and a short mild fever in less than 1/3 of women with acute infection [40]. Another study, carried out during the outbreak in Micronesia in 2007, found a similar symptom frequency, differing only in the fever incidence, which occurred in 65% of the cases [39].

### Chikungunya

Since its discovery, four different genotypes of the Chikungunya virus have been identified: East-Central-South-African (ECSA), West African, Asian and Indian Ocean lineage (IOL). The incubation period is usually from 3 to 7 days (ranging from 1 to 12 days) and 80% of the cases are symptomatic [41].

Fever is typically elevated, with a sudden onset, associated with arthralgia in almost all cases. Joint pain is usually symmetrical and can be disabling, more intense in the morning, improving with moderate exercise. The joints most often involved are the ankle, wrists and fingers. About 50% of the patients present a maculopapular rash and, occasionally, vesicular-bullous lesions and ulcers. Other symptoms include headache, myalgia, conjunctivitis, nausea and vomiting [42].

Symptoms typically resolve within 7 to 10 days, but some patients may still present rheumatologic symptoms for long periods after the initial infection.

### Dengue

Pregnant women are considered a risk group and are more likely to progress to more severe forms of the illness and death [43, 44].

Four serotypes of the Dengue virus exist: DENV-1, DENV-2, DENV-3 and DENV-4. Primary infection by one serotype confers immunity against reinfection by the homologous virus, but when infection by another serotype occurs, which is common in endemic areas, the risk of severe disease is increased due to a phenomenon known as antibody-dependent enhancement [45].

The incubation period varies from 4 to 10 days. About 50% of the infections are symptomatic and may present a broad clinical spectrum. The classification for dengue severity is divided into Dengue without warning signs, Dengue with warning signs, and severe Dengue. For the symptomatic patients three phases may occur:

1st phase - febrile, lasting from 2 to 7 days. The first manifestation is fever, usually high, associated with headaches, myalgia, arthralgia and retro-orbital pain. The rash, predominantly maculopapular, is present in 50% of the cases. Nausea and vomiting may be present. Most patients present improvements in their general state, but 5% progress to the critical phase.

2nd phase - critical, beginning with the defervescence of the fever, between the third and seventh day of the onset of the disease, accompanied by the appearance of one or more warning signs, including:

- Abdominal pain or tenderness;
- Persistent vomiting;
- Clinical fluid accumulation (i.e., pleural effusion or ascites);
- Mucosal bleeding;
- Lethargy or restlessness;
- Liver enlargement (≥2 cm);
- Increases in hematocrit concurrent with rapid decrease in platelet count.

3rd phase - recovery, lasting from 3 to 5 days, after which there is progressive clinical improvement of patients who have suffered the critical phase.

Severe Dengue is defined in the presence of one of the following criteria:

- Severe plasma leakage, leading to shock or fluid accumulation with respiratory distress;
- Severe bleeding;
- Severe organ involvement (AST or ALT ≥1000; impaired consciousness; failure of heart and other organs).

The process that determines the infection progression to severe forms appears to be multifactorial, involving individual immunological status and virus-related factors.

### Yellow fever

Like other infectious diseases, Yellow Fever has a wide spectrum of severity, ranging from asymptomatic patients to fatal cases.

The incubation period of the disease ranges from 3 to 6 days. Most symptomatic patients present with fever, headache, chills, muscle aches, and nausea, usually lasting a week. About 10 to 25% develop hemorrhagic manifestations and hepatic and renal impairment, with mortality occurring in 20 to 50% of the symptomatic cases [46].

The disease manifests itself in three stages: infection, remission and intoxication. In milder cases, patients recover without sequelae within a few days. In severe cases, patients develop a more severe form of the disease after the remission period, lasting from 24 to 48 h, called the intoxication period, and may present coagulopathy, haemorrhagic manifestations, jaundice, urinary albuminuria and acute renal failure [19].

## Fetal and neonatal outcomes

### Zika

A cohort of pregnant women with suspected Zika virus infections was carried out in Rio de Janeiro, between September 2015 and May 2016. Among the 125 pregnant women with confirmed diagnoses, 46.4% presented adverse pregnancy outcomes. Acute infection occurred between 6 and 39 weeks of gestation and complications were observed in 55% of cases when the infection occurred in the first trimester, 52% when infection occurred in the second trimester and 29% when the pregnant women were infected in the last trimester. Abortion cases accounted for 25% of first trimester outcomes and 3% of second trimester outcomes, with 2 fetal deaths among 34 pregnant women with a positive third trimester examination [40].

Of the 117 neonates, 42% displayed abnormal findings in the first month of life, with almost all cases presenting central nervous system abnormalities. Microcephaly was diagnosed in 3.4% of cases and restriction of fetal growth in 9%. The neurological abnormalities, similar to other studies [47, 48], included cerebral calcification, cerebral atrophy, ventriculomegaly, hypoplasia of the cerebral structures and hemorrhage of the cerebral parenchyma [40].

The neurological findings, observed even in late infections, result from central nervous system tropism of the virus, leading to the occurrence of fetal microcephaly and Guillain-Barré syndrome in adults [49, 50].

A previous case–control study, had already established the association of the Zika virus with the occurrence of fetal and neonatal neurological abnormalities. The analysis included 32 neonates with microcephaly, considered cases, and 62 neonates without microcephaly as controls, born in eight public maternity units in the metropolitan north-eastern region of Brazil. Eleven of 32 cases showed severe microcephaly and significantly higher proportions of cases compared to controls were born with low birth weight and were small for their gestational age. Of the 27 cases investigated by brain imaging, 11 presented one or more abnormalities [51].

Another study analyzed the data extracted from the epidemiologic reports published by the Secretary of Health Surveillence of the Brazilian Ministry of Health, that monitors cases of microcephaly associated with congenital infection in Brazil. Considering the confirmed cases of microcephaly and related deaths associated with Zika virus in Brazil, from November 2015 to October 2016, the estimated case fatality rate is 8.3% [52].

### Chikungunya

Maternal-fetal transmission occurs mainly when there is maternal viremia at the time of delivery, with onset of neonatal symptoms 3 to 9 days after birth.

The main clinical manifestations in symptomatic newborns are fever, irritability, exanthema, lower or generalized lower limb edema, hyperalgesia, vesicular-bullous dermatosis, meningoencephalitis and hemodynamic instability [35]. Some cases may progress with neonatal encephalopathy and microcephaly.

A 2-year follow-up of a cohort of children exposed to the virus during the perinatal period showed a delay in global neurodevelopment, with low developmental rates of those exposed compared to negative controls for infections [53].

### Dengue

Two possible mechanisms may be responsible for fetal and neonatal morbidity due to dengue virus infection during pregnancy: the presence of maternal hemodynamic changes, which may compromise the placenta and cause fetal hypoxia, and the direct effect of infection on the fetus.

Paixão et al. conducted a systematic review and meta-analysis of adverse fetal outcomes resulting from Dengue infection during gestation. Sixteen studies were included in the systematic review and eight were eligible for the meta-analysis, totaling 292 women exposed to the Dengue virus during gestation. The review indicated an association of Dengue in pregnancy with abortion (OR 3.51), fetal death (RR 6.7), preterm birth (OR 1.71) and low birth weight (OR 1.41) [54]. Other studies have verified similar results, especially with regard to prematurity and low birth weight [55–58].

Ribeiro et al. studied the fetal outcome of 24 pregnant women with laboratory-confirmed Dengue infections and observed an increase in the incidence of abortions (20,8%), fetal death (8.3%) and prematurity (12.5%). After birth, 29% of the newborns presented symptoms of the disease [59].

The Dengue virus is endemic in many localities and the prevalence of infection during pregnancy, with consequent fetal impairment, may vary according to the local scenario and the cumulative period of exposure to the virus [43, 60]. The analysis of the prevalence and incidence of Dengue during pregnancy in a great epidemic in the central region of Brazil indicated an incidence of 2.8%, diagnosed through the presence of maternal IgM and a prevalence of 53.9% identified by maternal IgG. When umbilical cord blood was analyzed in IgG positive pregnant women, the transfer of maternal antibodies to the fetus was observed to be 99.3% [55].

### Yellow fever

Most studies regarding Yellow Fever analyzed fetal and neonatal effects after inadvertent vaccinations during pregnancy.

In 2009, a case of perinatal transmission of Yellow Fever was described during an epidemic in the state of São Paulo, Brazil. The 30-year-old woman presented with fever, headache and jaundice 3 days before vaginal delivery. The female newborn was discharged after 2 days and on the third day was readmitted with fever and cyanosis, progressing with hemorrhage, liver enzyme alterations, oliguria and death with 12 days of life. The neonate underwent antibiotic therapy and intensive support during hospitalization. Confirmation of newborn infection was obtained by RT-PCR harvested after 5 days of the onset of symptoms. The maternal infection was confirmed by IgM positive for Yellow Fever and negative for Dengue [38].

## Diagnoses

### Clinical presentation

Due to the similarity between these arboviruses symptoms and the possibility of rapid evolution to severe forms, it is recommended that infection by the Dengue virus be considered as the main differential diagnosis in patients who have been in endemic areas. Infections with the Zika and Chikungunya virus should also be considered, since they present the same vector and many regions are endemic for different arboviruses.

Table 1, released by the Fiocruz Agency, summarizes the characteristics and frequencies of symptoms in Dengue, Zika and Chikungunya.

**Table 1:** Comparison of symptoms of symptomatic presentation^a^ between Dengue, Zika and Chikungunya

Signs/Symptoms	Dengue	Zika	Chikungunya
Fever	Above 38 °C (4 to 7 days)	No fever or subfebrile ≤38 °C (1 to 2 days)	Above 38 °C (2 to 3 days)
Rash	Appears from the 4th day 30–50% of the cases	Appears on the 1st or 2nd day 90–100% of cases	Appears on the 2nd or 5th day 50% of cases
Myalgia	+++/+++	++/+++	+/+++
Arthralgia	+/+++	++/+++	+++/+++
Conjunctivitis	Rare	50–90% of cases	30%
Headache	+++	++	++
Pruritus	Slight	Moderate/Intense	Slight
Blood dyscrasia	Moderate	Absent	Slight
Neurological impairment	Rare	More frequent than Dengue and Chikungunya	Rare (predominant in neonates)

^a^Estimated symptomatic presentation in 50% of Dengue virus, 20% of Zika virus and 80% of Chikungunya virus infection

### Laboratory tests

Some endemic areas display multiple arboviruses which present the same symptoms, such as fever, rash, myalgia, and arthralgia. Therefore, laboratory evaluation of the Dengue, Zika and Chikungunya viruses, as well as other TORCH group diseases (Toxoplasmosis, Rubella, Cytomegalovirus and Herpes) is recommended to establish the diagnosis.

In the acute phase, which occurs within the first 7 days after the onset of the symptoms, virus RNA can be identified in the patient’s serum, with RT-PCR as the test of choice for the diagnosis of Dengue, Zika and Chikungunya. In the case of Dengue infection, the NS1 antigen can also be detected in the acute phase by the ELISA test. However, this test is not widely available. A negative result, however, does not exclude the infection, and a serological test for IgM antibodies is necessary.

In the convalescence phase, the ELISA test for IgM antibodies, which are usually detectable between 2 and 12 weeks after infection, should be performed for Dengue, Zika and Chikungunya. However, due to cross-reactions, a positive serology (IgM) for Dengue or Zika only indicates a recent flavivirus infection and it is not possible to distinguish the type of virus in question. In case of positive IgM antibodies, the Plaque Reduction Neutralization Test (PRNT), which characterizes and quantifies circulating levels of anti-Dengue virus neutralizing antibody, is necessary to confirm the diagnosis. Patients who have received a Yellow Fever vaccine in the past may also present serology cross-reactivity [61, 62].

The Trioplex RT-PCR is a laboratory test designed to detect Zika, Dengue and Chikungunya virus RNA, authorized by the FDA to be used under an Emergency Use Authorization (USA) [63].

The diagnosis of a Yellow Fever infection follows the same protocol with RT-PCR, and the detection of the NS1 antigen with high sensitivity and specificity, possible during the acute phase. The identification of Yellow Fever antibodies by IgM ELISA can also be performed. Serology assays, however, present limitations due to the possibility of cross-reactivity with other flaviviruses [64].

In addition to serum, RT-PCR can also be performed on urine and saliva samples. Musso et al. analyzed saliva samples from symptomatic patients during the French Polynesia epidemic from October 2013 to March 2014, and, although there was no increase in the virus detection window, the saliva sample increased the molecular detection rate of viral RNA [65].

## Treatment

There is no specific medication for infections caused by Dengue, Zika, Chikungunya or Yellow Fever viruses. The treatment consists in rest, drinking fluids to prevent dehydration and the use of analgesics, such as acetaminophen. In the presence of warning signs (section Dengue), the patients must be admitted to a hospital where they can receive intensive care. Gestation, since it is considered a risk condition for Dengue infection, should present a follow-up in the maternity hospital or health unit.

The use of aspirin or other non-steroidal anti-inflammatory drugs must be avoided. The use of these medicines may aggravate the hemorrhagic condition in case of Dengue virus infection [66].

## Prevention

### Vaccines

The Dengue vaccine CYD-TDV has recently been licensed but still remains largely unused. It is a tetravalent live attenuated viral vaccine, licensed for the prevention of Dengue illness caused by Dengue virus serotypes 1, 2, 3 and 4 in individuals ranging from 9 to 45 years old or 9 to 60 years old, depending on the license, living in Dengue endemic areas. The vaccination schedule consists of 3 0,5 mL injections administered at 6-month intervals, with an average efficiency of approximately 60%.

There is no recommendation concerning CYD-TDV during pregnancy and breastfeeding, due to lack of sufficient data in this population. However, the limited data collected during the clinical trials on unintentional immunization in pregnancy have yielded no evidence of harm to the fetus or pregnant women. To date, no studies evaluating the effects of inadvertent use in pregnant women are available [67].

Yellow Fever 17D vaccine is widely used for the prevention of Yellow Fever in travelers, for routine immunization in endemic areas and for emergency response during outbreaks. It is given in a volume of 0,5 ml by the subcutaneous route in a dose of no less than 1000 International Units. Approximately 90% of vaccines seroconvert by 10 days and 99% seroconvert by 30 days post immunization.

The Yellow Fever vaccine is not recommended during gestation or breastfeeding, since it is composed of a live attenuated virus, except in situations of a severe epidemic or unavoidable trip to high-risk locations [68]. However, studies analyzing Yellow Fever vaccination in pregnant women found no increased risk of adverse events in gestation and newborns, except in a study that presented insufficient statistical power [69–73]. The risk of miscarriage, malformations, fetal death and prematurity is similar to that of the general population [74]. It is noteworthy that many of these studies reported small series.

If vaccination is required during pregnancy, it is preferable to be administered during the first trimester, when the immune response appears to be more satisfactory. The vaccine may be administered during breastfeeding, which should be discontinued for 10 days during the post-vaccine viremia phase. Regarding breastfeeding, a case of vaccine-induced encephalitis in one infant was reported 8 days after the mother’s primary vaccination [75].

At the time of publication of this article, no vaccines against Zika and Chikungunya were available. Future vaccine programs should consider the serological cross-reaction between the Zika virus and Dengue virus and the implications for disease pathogenesis [76].

### Vector control

The preventive strategy considered most efficient is vector control, through the use of repellents and long-sleeved clothes, placement of screens in houses, sleeping in rooms with air conditioning and avoiding the accumulation of standing water, which serves as a breeding ground for the mosquito vectors. The WHO recommends the use of repellents containing DEET, IR3535, aminopropionic or icaridine [77].

### Other measures during pregnancy

The CDC recommends sexual abstinence or condom use for men suspected of contracting a Zika virus infection over a period of 6 months. For those residing or returning from a region with active transmission of the virus but who do not show symptoms, such measures should be adopted for at least 8 weeks after the person’s return.

Women who are interested in childbearing and pregnant women should not travel to areas with the risk of Zika. If travel is unavoidable, or with regard to pregnant couples living in Zika areas, condoms from start to finish every time sex is performed is recommended, during the entire pregnancy. Populations in endemic areas or who have been exposed should conduct early and serial ultrasounds during pregnancy [78].

## Conclusions

Arboviruses are emerging infectious diseases endemic in several localities, but with unpredictable and devastating potential, as seen recently with the viral epidemics in the Americas.

Despite the recent publications on infections by the Zika virus and the repercussions of congenital infection, research in this area is still modest and many arboviruses aspects need to be clarified, especially regarding gestational infection.

Some issues that should be focused on are the improvement of diagnostic tests, including specific and sensitive molecular tests, associated to ultrasound screening; more accurate estimates of maternal, fetal and neonatal risks and the development of preventive vaccines, among other therapeutic options.

Only after the advancement of these issues will it be possible to develop effective intervention strategies aimed at the prevention or eradication of arbovirus infections and their deleterious maternal-fetal outcomes.
